# Innate iNKT cells: from biological insight to clinical impact

**DOI:** 10.3389/fimmu.2025.1653183

**Published:** 2025-09-10

**Authors:** Antonia Rotolo, Nicola J. Mason, Mark A. Exley

**Affiliations:** ^1^ Department of Pathobiology, School of Veterinary Medicine, University of Pennsylvania, Philadelphia, PA, United States; ^2^ Center for Cellular Immunotherapies, Perelman School of Medicine, University of Pennsylvania, Philadelphia, PA, United States; ^3^ Brigham & Women’s Hospital, Mass General Brigham, Boston, MA, United States; ^4^ Gastroenterology, MiNK Therapeutics, Lexington, MA, United States; ^5^ Life Sciences, Imperial College, London, United Kingdom

**Keywords:** CD1, CD1d, invariant natural killer T (iNKT) cell, NKT cell, allogeneic cells, off-the-shelf cells, graft versus host disease (GVHD), COVID-19

## Abstract

Over the past 30 years, work of immunologists worldwide has phenotypically and functionally defined “Natural Killer T cells” (NKT) and their subsets, including “invariant Natural Killer T cells” (iNKT). NKT cells make up a substantial fraction of T cells that express NK cell markers and have TCRs restricted to either conventional MHC molecules or the monomorphic CD1d molecule. Among these, iNKT cells are CD1d-restricted and more common within NKT cells than T cells without NK markers. While the definition of NKT cells, whether based on phenotype, function, or both, remains a topic of debate, iNKT cells represent a distinct T cell population characterized by a recurrent, conserved TCR rearrangement (TRAV10–TRAJ18 in humans) paired with a limited Vβ repertoire (mostly encoded by TRBV25-1 in humans). iNKT cells are restricted by CD1d, which, unlike CD1a-c molecules, is expressed not only on professional antigen-presenting cells and thymocytes but also on certain non-hematopoietic somatic tissues, both normal and neoplastic. Like all CD1 family members, CD1d presents various lipid antigens by accommodating their long hydrophobic tails in deep binding pockets, in contrast to the shallow peptide grooves of conventional MHC molecules. However, the ligand repertoire of CD1d is distinct from that of CD1a-c. This review focuses on CD1d-restricted iNKT cells. Activation of iNKT cells via their semi-invariant TCR, often in synergy with NK receptors and other co-stimulatory molecules, triggers a rapid, polyfunctional response. Unlike conventional MHC-restricted T cells, individual iNKT cells can simultaneously produce both Th1- and Th2-type cytokines and exert cytotoxic activity in an immune synapse-directed fashion. Through this combination of direct cytotoxicity and cytokine-mediated immunomodulation, iNKTs can eliminate target cells while activating myeloid and other lymphoid populations to amplify immune responses. Their versatility has fueled growing interest in harnessing iNKT cells across inflammatory, infectious, and oncological diseases, where early-phase studies have demonstrated their safety and preliminary efficacy. Moreover, because they are restricted by the non-polymorphic CD1d molecule and possess immune-regulatory properties, iNKT cells lack graft-versus-host potential, making them ideal candidates for allogeneic, off-the-shelf therapies. This review summarizes how iNKT cells are being reimagined as innovative tools for immune intervention across a range of clinical settings.

## 1 Introduction: iNKT cells act as hybrid immune powerhouses

Invariant Natural Killer T (iNKT) cells are a unique, rare subset of T cells (0.01%-0.2% on average) (1–3) that play crucial roles in immune regulation, homeostasis and defense against pathogens and cancers (4–10). They are termed “invariant” because they express a highly conserved T cell receptor (TCR), characterized by an α-chain with a nearly constant junction across individuals (TRAV10–TRAJ18 in humans), paired with a β-chain that uses a limited set of Vβ genes (mostly TRBV25-1 in humans), combined with diverse D and J gene segments. While the TCRα chains are formally oligoclonal rather than monoclonal, they typically encode an identical amino acid sequence through different codon combinations, and are highly conserved across species, from mice to humans (11).

The story of iNKTs began in the late 1980s, when researchers discovered unusual lymphoid cells with a hybrid T and NK cell phenotype in mice (12). These cells predominantly expressed αβ TCRs along with CD161, then known as NK1.1 in mice and NKR-P1A in humans, an antigen previously thought to be exclusive to NK cells. This hybrid identity led to their initial designation as “NK T cells” in 1995 (12). In parallel, a population with an almost clonal TCRα and a limited set of TCRβ chains was described in both humans and mice (5, 6). Bendelac and colleagues showed that these cells were restricted to the monomorphic, MHC-related molecule CD1d, linking them to a subset of the previously described “NK T” cells in mice, and revealing these cells to represent one and the same population rather than two separate ones (12, 13). Their human counterparts were reported two years later by Exley and colleagues (14).

What truly sets iNKTs apart from conventional T cells is their invariant TCR α-chain, in contrast to the diverse TCRs expressed by the latter (15). This discovery led to the introduction of the term “invariant NKT cells” or “iNKT cells” by S. Brian Wilson and his team in 2001 (16). Researchers also realized that activation and development of these cells required β2-microglobulin, even though the majority do not express the CD8 co-receptor and were either CD4^+^ or ‘double-negative’ (DN) for both CD4 and CD8 (4, 17–21). This finding helped establish iNKT cells as a unique subset of T cells with specific mechanisms of function and a distinct, specialized role in the immune system.

**Figure 1:**
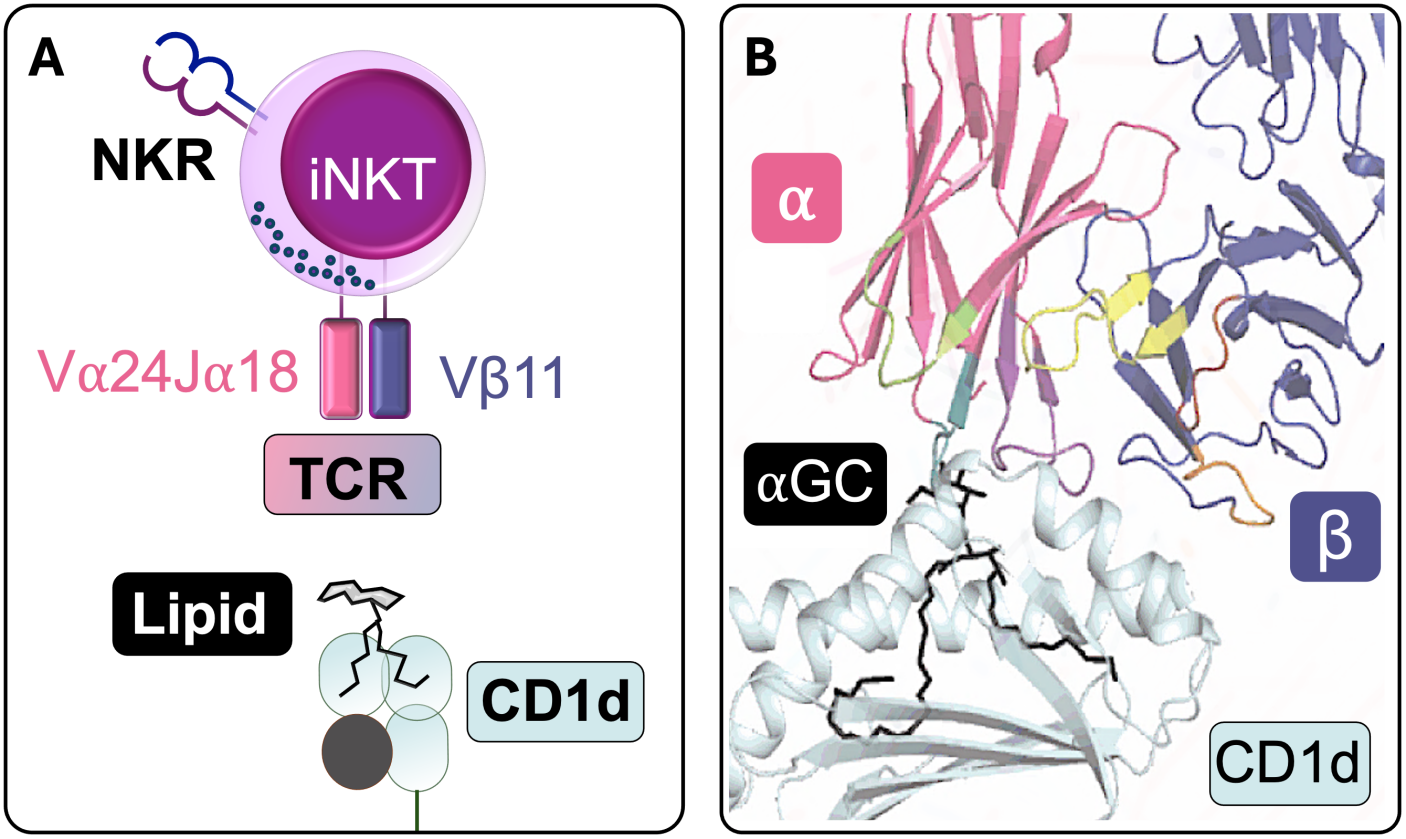
iNKTCR recognition of CD1d-lipid. **(A)** Schematic representation of a human iNKT cell recognizing a CD1d-lipid antigen complex (NKR: NK receptor; TCR: T cell receptor). **(B)** Structural depiction of stereospecific recognition of α-galactosylceramide (αGC) in complex with CD1d. The lipid tails are buried within CD1d’s hydrophobic pocket, whereas the polar headgroup protrudes from CD1d to interact with the TCR. The TCRα chain makes contact with αGC, whereas the β chain primarily interacts with CD1d, contributing to the modulation of overall TCR affinity (Adapted from Rossjohn J., et al, 2015 (141)).

The identification of CD1d as the restriction element for iNKT cells was a major functional breakthrough (13). Of note, while mice and rats only have one or two CD1d genes (6, 17), multiple isoforms, namely, CD1a, CD1b, CD1c, and CD1e, were identified in humans, with CD1e remaining intracellular. Soon after, it became clear that CD1d, like the other CD1 isoforms, presents lipid antigens derived from both endogenous and microbial sources (22–25). CD1d is found on antigen-presenting cells (APCs), including B lymphocytes, monocytes, dendritic cells (DCs) and macrophages, as well as thymocytes and certain non-hematopoietic cells such as epithelial, parenchymal and vascular smooth muscle cells (26–28). Because of the invariant TCRα and its peculiar binding properties, iNKTs are uniquely adept at stereospecific recognition of α-galactosylceramide (αGalCer) in complex with CD1d (29) (**Figure 1**), which drives potent expansion of human iNKTs (30). The development of CD1d tetramers loaded with αGalCer enabled direct detection of these cells and solidified their classification as a highly specialized T cell subset (31, 32).

Today, iNKTs are recognized for their unique biological features, which hold great promise for developing novel cellular therapies to treat challenging conditions such as cancer, infections, and inflammatory diseases, conditions that are often difficult or unsuitable to address with conventional MHC-restricted T and NK cells.

In this review, we discuss the latest advances in translating iNKT therapeutic potential into clinical practice.

## 2 iNKT cells have wide therapeutic potential across individuals and disease settings

iNKT cells are a rare yet strikingly heterogeneous T cell subset, comprising functionally specialized subpopulations. Historically, CD4^-^ and CD4^+^ subsets have been used to distinguish human iNKT populations with enhanced cytotoxic potential versus those with greater regulatory capacity respectively (33). Subsequent studies have revealed broader human iNKT diversity by combining expression of canonical surface markers of T and NK activation, differentiation and homing (e.g., CD28, CD27, CD62L, NKG2D, CD161, KLRs, CCR7, CCR5) (14, 21, 34, 35), with human iNKT-specific cytokine patterns and transcriptional programs (36,37). Unlike their mouse counterparts, human iNKT subsets are less clearly defined, consistent with their marked polyfunctionality. Furthermore, although they share classical memory and effector molecules with MHC-restricted T and NK cells, human iNKTs cannot be readily classified into conventional naïve, memory, and effector states (38). Still, their ability to proliferate, survive, and function varies both within and across individuals, correlating with CD62L^+^ phenotypic patterns (34) and with exposure to distinct cytokine milieus (39, 40), differences that may lead to divergent functional outcomes in therapeutic settings. While a detailed description of iNKT subset biology is beyond the scope of this review, these insights underscore that the therapeutic potential of human iNKT cells lies not only in their unique biology but also in their heterogeneity. Decoding iNKT diversity is an active area of research, critical to understanding how best to deploy iNKT cells in the clinic, for instance, to predict treatment responses or to design tailored strategies targeting specific tissues or modulating the duration and nature of immune responses (e.g., cytotoxic, immunoadjuvant, or tolerogenic). This is especially relevant for the development of allogeneic and off-the-shelf iNKT-based cell therapies, where pre-manufactured products could be customized to match desired immune outcomes.

**Figure 2:**
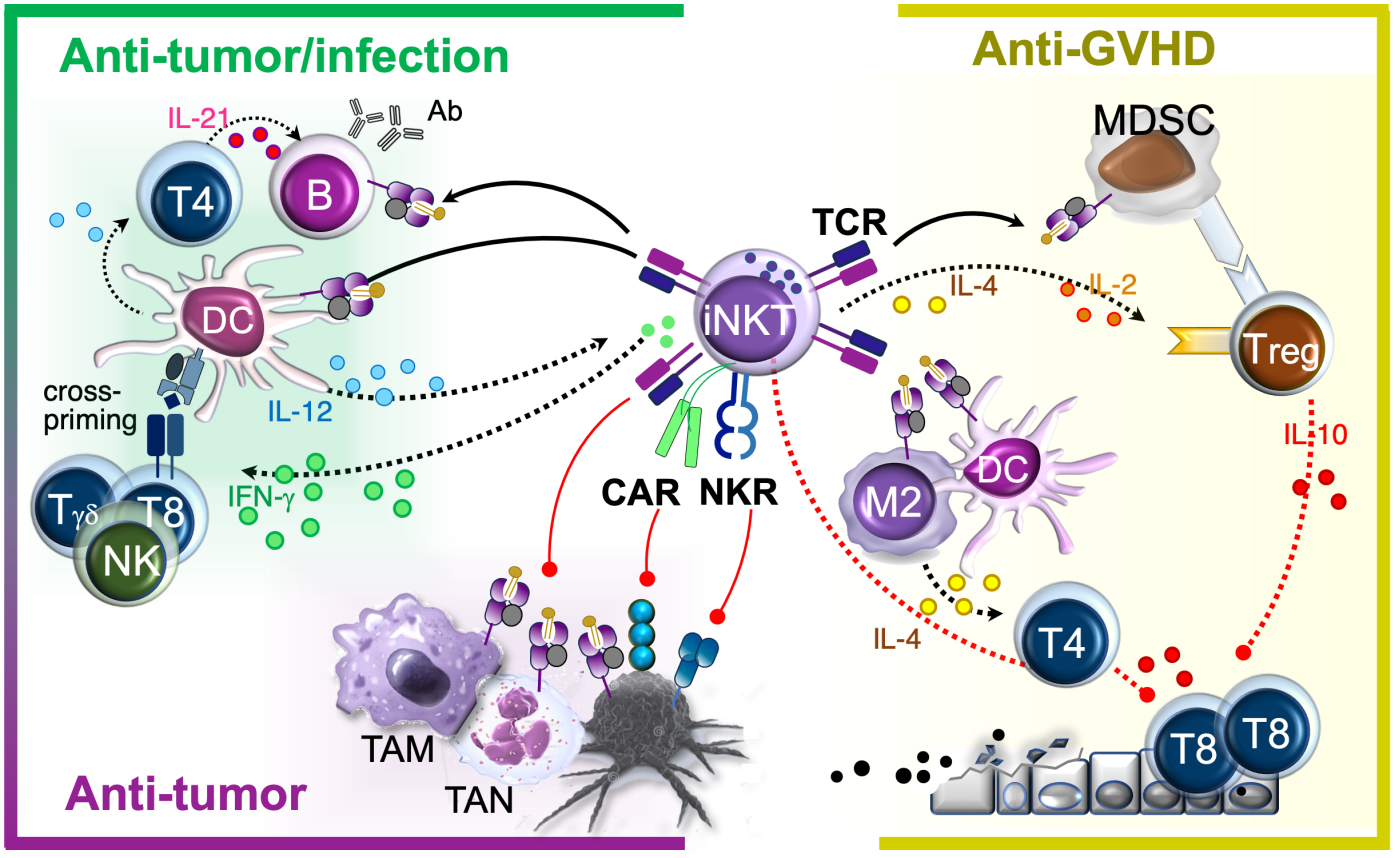
iNKT therapeutic effects. iNKTs naturally home to infection and tumor sites where they activate APCs and recruit endogenous immune cells, playing a critical role in reprogramming the microenvironment to promote antimicrobial and tumor-directed immunoadjuvant responses. In parallel, they exert direct effector functions by targeting both immunosuppressive cells within the microenvironment and infected or malignant cells, contributing to disease eradication. These immune-activating functions coexist with the selective suppression of alloreactive cells in allogeneic settings. In immunocompetent murine models, such effect was attributed to the induction of immunomodulators (MDSC, Treg, Th2 cells). In human xenograft models, allosuppression was also mediated by the direct elimination of APCs capable of activating alloreactive T cells. *Black arrows:* activation. *Red vectors*: inhibition. *Solid lines*: contact-mediated effects. *Dotted lines*: cytokine-mediated effects. TAM, Tumor-Associated Macrophage; TAN, Tumor-Associated Neutrophil; M2, M2 Macrophage; T4, CD4^+^ T cell; T8, CD8^+^ T cells.

Owing to their ability to both modulate immune responses and act directly as effector cells, iNKTs are emerging as powerful players in the immune system, showing protective roles in cancer (41), infections (42, 43), and immune-mediated conditions (44). Further, they combine features of both innate and adaptive immunity, which makes them ‘hybrid effectors’ with a primed ‘ready-to-act’ state (45). Much like classical innate immune cells, iNKTs respond rapidly to danger signals, pro-inflammatory cytokines, and CD1d-presented lipid antigen stimuli within minutes or hours. At the same time, like conventional MHC-restricted T cells, they use their TCR to initiate specific, CD1d-restricted responses, including cytotoxicity and immune modulation (46–49). Though relatively rare, iNKT cells compare favorably with antigen-specific T cells in effector and immunomodulatory functions. This along with their innate-like reactivity allows iNKTs to both act faster than T cells and produce unusually large amounts of cytokines per cell that orchestrate both innate and adaptive immune responses (**Figure 2**,left). As a result, iNKTs have significant therapeutic potential that extends beyond cancer immunotherapy, offering hope for treating severe infections and chronic inflammatory conditions.

One of the main iNKT advantages for therapy is their ability to be transferred from donor to recipient without causing graft-versus-host disease (GVHD) (50–52). This property stems from their invariant TCRα chain and restriction to the monomorphic CD1d molecule, and is in stark contrast to classical MHC-restricted T cells, eliminating the need for endogenous TCR ablation required for allogeneic conventional T cells. As a result, banks of allogeneic iNKT cells can be generated for off-the-shelf cellular therapy, circumventing common T cell therapy challenges related to T cell fitness, manufacturing costs and time needed to generate autologous T cell products from patient apheresis or heavily gene edited allogeneic products from healthy donors. In addition to avoiding GVHD, iNKT cells can suppress alloreactive donor T cells that cause GVHD, as part of their specialized ability to orchestrate immune responses (53–59) (**Figure 2**, right). This property is particularly valuable in allogeneic hematopoietic stem cell transplantation (HSCT), where iNKT cells can help prevent and control potentially lethal immune responses while simultaneously promoting protective immunity through graft-versus-tumor (GVT) effects (53), and anti-microbial defense (60). These functions are especially critical for HSCT recipients, who are typically immunosuppressed due to their underlying disease, conditioning regimens and prolonged anti-GVHD therapies. Additionally, by suppressing inflammatory triggers known to impair hematopoietic stem and progenitor cell engraftment (61), iNKTs can support durable donor chimerism without requiring complete elimination of host immune cells (61). This whole-in-one functionality has the potential to extend graft persistence (61) while minimizing alloreactive flare-ups (55, 56), infectious complications (60) and disease recurrence (53) thereby enhancing overall transplant success. Altogether, these seemingly paradoxical actions, i.e., suppressing harmful immune responses while enhancing protective ones, make context-reactive iNKT cells valuable for a wide range of applications in transplantation and allogeneic cell therapies, both as unmodified (50, 51, 62) and engineered products for enhanced therapeutic benefit (52).

Lastly, iNKTs exhibit pronounced tissue tropism and home to specific tissues more effectively than circulating conventional MHC-restricted T cells, displaying tissue-resident-like behaviors, driven by distinct chemokine and homing receptors expression patterns (63, 64). In mice, the anatomical distribution of iNKTs is well correlated with functional specialization and is amenable to pharmacologic modulation (65, 66). For instance, intravenous administration of αGalCer activates hepatic and splenic perivascular iNKTs, inducing robust systemic IFN-γ responses, whereas oral delivery primarily engages iNKTs in mesenteric lymph nodes, triggering localized IL-4-mediated tolerogenic effects (66). Similarly, in humans, emerging evidence points to tissue-specific functional programs that could be leveraged to shape therapeutic responses in cancer, vaccine, and transplant settings, modulating the type (immunoadjuvant or tolerogenic), extent (systemic or local) and kinetics (rapid or delayed) of iNKT-cell based immunotherapies. Importantly, tissue homing capacities have been linked to increased trafficking and function at tumor sites (67–69), where iNKTs can play a critical role in switching the tumor microenvironment (TME) from immunosuppressive to pro-inflammatory and anti-tumor. In fact, by targeting immune-suppressive myeloid cells via CD1d, recruiting cytotoxic T and NK cells, and activating APCs, iNKTs can create the conditions necessary for tumor eradication in solid malignancies and lymphoproliferative solid-like neoplasms (64, 67, 69–71). Peripheral blood-derived iNKTs are practical to isolate and broadly reflect features of their tissue-resident counterparts, supporting their use as versatile effectors capable of trafficking to and functioning within diverse tissues, including immune-privileged sites such as the brain. However, certain iNKT subsets may exhibit superior trafficking or persistence in specific anatomical niches. Disease-specific applications could therefore benefit from the enrichment or use of tissue-derived iNKTs, analogous to tumor-infiltrating lymphocyte (TIL) therapies, to enhance site-specific activity. Accordingly, therapeutic strategies may need to account for tissue-specific differences when designing persistence-tracking approaches, including consideration of tumor biopsies or bone marrow sampling, depending on the disease context.

In conclusion, this collection of features establishes iNKTs as a highly versatile T cell subset with broad therapeutic potential. Their ability to simultaneously exert effector functions, modulate immune responses, facilitate donor cell/tissue engraftment, and home to specific sites, combined with a safe therapeutic profile, makes them a promising tool, not just for cancer immunotherapy, but also for treating severe infections, systemic inflammatory diseases, and enhancing outcomes in hemopoietic and organ/cell transplantation. Their role in tissue immune responses and homeostasis also opens up new opportunities to address challenges that conventional T and NK cell-based therapies still face (72, 73).

## 3 iNKT cells can induce complete remissions in solid cancer patients

Tumors arising from certain CD1d^+^ cell types, such as some B cell lymphomas, commonly retain CD1d expression, though levels may vary with tumor stage, for example, declining during progression in multiple myeloma (74). However, in cancer patients, iNKT frequency and/or function are often diminished (75–81). This reduction in both the number of circulating iNKTs and their ability to function effectively is associated with poorer clinical outcomes (reviewed in (82, 83)). Likewise, both basally and therapeutically increased iNKT numbers have been linked to better prognoses and improved patient outcomes (80, 84). Collectively, these observations suggest that patient’s own iNKT cells play a meaningful role in anti-tumor immunity, supporting strategies that aim to restore the number and function of endogenous iNKT cells.

Importantly, patient-derived iNKT cells can be expanded *ex vivo*, and show repaired functionality under optimal culture conditions, reversing the dysfunction seen in cancer patients (75, 78–80, 85). Expanded autologous iNKTs retain their natural ability to home to tumors and mitigate tumor-associated immune suppression and heterogeneity through direct anti-TME activity and NK-like cytotoxicity, alongside immunoadjuvant effects (see below). These findings support the use of iNKT-based therapies, including patient-derived expanded iNKTs, to enhance endogenous iNKT numbers and function. Whether through endogenous activation or adoptive transfer, autologous iNKT cells offer a valuable platform to restore anti-tumor immunity and provide a novel therapeutic strategy for cancer patients (**Table 1**).

**Table 1:** Autologous iNKT trials.

Trial ID	Disease	iNKT therapy	Pre-conditioning	Patient Number	Best Response (number of patients)	Other responses (number of patients)	References
Monotherapy							
NA	NSCLC	iNKT-enriched PBMCs	No	6	SD (2)	NA	(86)
NA	Melanoma	iNKT	No	9	SD (6)	NA	(87)
NCT03294954	NB	IL15.CAR-GD2 iNKT, autologous	Flu/Cy	12	CR (1)	PR (2)	(98, 99)
NCT06182735	RCC	CAR-CD70 iNKT, autologous	Flu/Cy	6	SD (2/4*)	NA	(101)
NCT06728189	Solid tumors	CAR-CD70 iNKT, autologous	Flu/Cy	NA	NA	NA	NA
NCT06394622	Solid tumors	CAR-CD70 iNKT, autologous	Flu/Cy	NA	NA	NA	NA
Combinations							
UMIN000000722	HNSCC	KRN7000-pulsed DC plus iNKT	No	8	PR (3)	SD (4)	(125)
UMIN000000852	HNSCC	KRN7000-pulsed DC plus iNKT	No	10	PR (5)	SD (5)	(126)

NA, Not Available; SD, stable disease; PR, partial response; CR, complete response; NSCLC, Non-small Cell Lung Cancer; NB, Neuroblastoma; RCC, Renal Cell Carcinoma; HNSCC, Head and Neck Squamous Cell Carcinoma; Flu/Cy, Fludarabine/Cyclophosphamide; *4 out of 6 patients were evaluable at the time of the report.

Early clinical trials in patients with lung cancer and melanoma explored the feasibility of expanding autologous (patient-derived) iNKTs *ex vivo* and re-infusing them into the same patients to restore iNKT’s normal frequency and functionality. In a study of six patients with non-small cell lung cancer (NSCLC), autologous peripheral blood mononuclear cells (PBMCs) were enriched for iNKTs over a 3-week expansion period and administered in two infusions at doses of 1 to 5 × 10^7^ iNKTs/m2/infusion (purity min – max: 0.01 - 25%). While the treatment did not induce tumor regression, it stabilized the disease in two patients for 9 and 12 months, with all participants showing signs of immune activation (86). A trial in melanoma patients employed an improved manufacturing protocol, entailing iNKT enrichment with a monoclonal antibody to the iNKT cell receptor (iNKTCR) prior to *ex vivo* expansion, resulting in higher purity of the autologous iNKT products (13- 87%, median 66%). Three out of nine patients remained progression-free for over four years (53-65 months), and the overall time to progression was approximately one and a half years, even including one patient with actively progressing disease at the time of treatment. Collectively, this pioneering experience provided encouraging data supporting the exploration of optimized iNKT strategies to further enhance clinical efficacy (87).

One promising approach to improve therapeutic success of iNKT cells involves genetic engineering with chimeric antigen receptors (CARs), recombinant TCRs (rTCRs), chimeric TCRs, and other synthetic modifications. In preclinical studies, CAR-iNKTs and rTCR-iNKTs were more effective than unmodified iNKTs in killing target cells, while retaining their innate trafficking and immunomodulatory capabilities alongside functionality of their endogenous TCR and NK receptors, required for maximal activity against the TME as well as malignant cells (34, 67, 88–97). In a landmark clinical trial (NCT03294954), twelve children and young adults with neuroblastoma unresponsive to the standard of care were treated with autologous iNKTs engineered to express a GD2-specific CAR and IL-15 (98, 99). This CAR-iNKT cell therapy proved safe and clinically effective, yielding one complete and two partial responses, alongside evidence of ***in vivo*** expansion and tumor homing (98). Of note, neuroblastoma patients treated at the same institution with third-generation GD2-specific CAR T cells, which are designed to be more potent than commonly used second-generation versions by including two costimulatory domains instead of one to enhance anti-tumor responses, did not achieve an objective response (100). Collectively, these results underscore a promising improvement in iNKT therapeutic activity that was enhanced via synthetic engineering. Another two ongoing trials (NCT06182735 and NCT06728189) are evaluating autologous CD70-targeted CAR-iNKT in patients with metastatic renal cell carcinoma (mRCC) and advanced solid tumors. Interim results from six mRCC patients revealed significant tumor reductions in two of four evaluable cases, with responses lasting at least nine months despite low CD70 expression (101). CAR-iNKTs persisted in circulation for five months, with no evidence of severe cytokine release syndrome (CRS) or immune effector cell-associated neurotoxicity syndrome (ICANS). These early data further corroborate the feasibility of genetically redirected iNKTs for the treatment of as-yet incurable solid cancers with promising signs of therapeutic activity. Further refinements are underway, and some are already being tested in the clinic, including innovative combinatorial strategies to optimize iNKT cell expansion, persistence, and anti-tumor potency in patients with solid cancers (see NCT03294954 below and **Table 1**). Additional pre-clinical efforts in partnerships with biotech companies are actively focusing on developing CAR-iNKT therapeutic products with desirable traits, such as less differentiated (CD62L^+^) (98), less exhausted (BTG1-knockdown) (98), and Th1-polarized subsets (40, 102), to enhance durability and functionality. Specifically, there is robust data supporting that CD62L expression marks a central memory-like population characterized by sustained anti-tumor effects in preclinical ***in vivo*** models (34) and more durable responses in patients (98, 99). This subset is especially attractive for therapeutic development, offering the potential for long-term immune surveillance and tumor control.

Taken together, these advancements and the promising clinical activity of CAR-iNKTs in challenging solid tumor patients mark a significant milestone in the cell therapy field. Beyond establishing the feasibility of this versatile cellular platform, these findings demonstrate that CAR-iNKTs can drive meaningful clinical responses, warranting further investigation for treating incurable diseases where existing therapies remain largely ineffective.

## 4 Allogeneic, off-the-shelf iNKT therapies are feasible in refractory cancer patients

**Figure 3:**
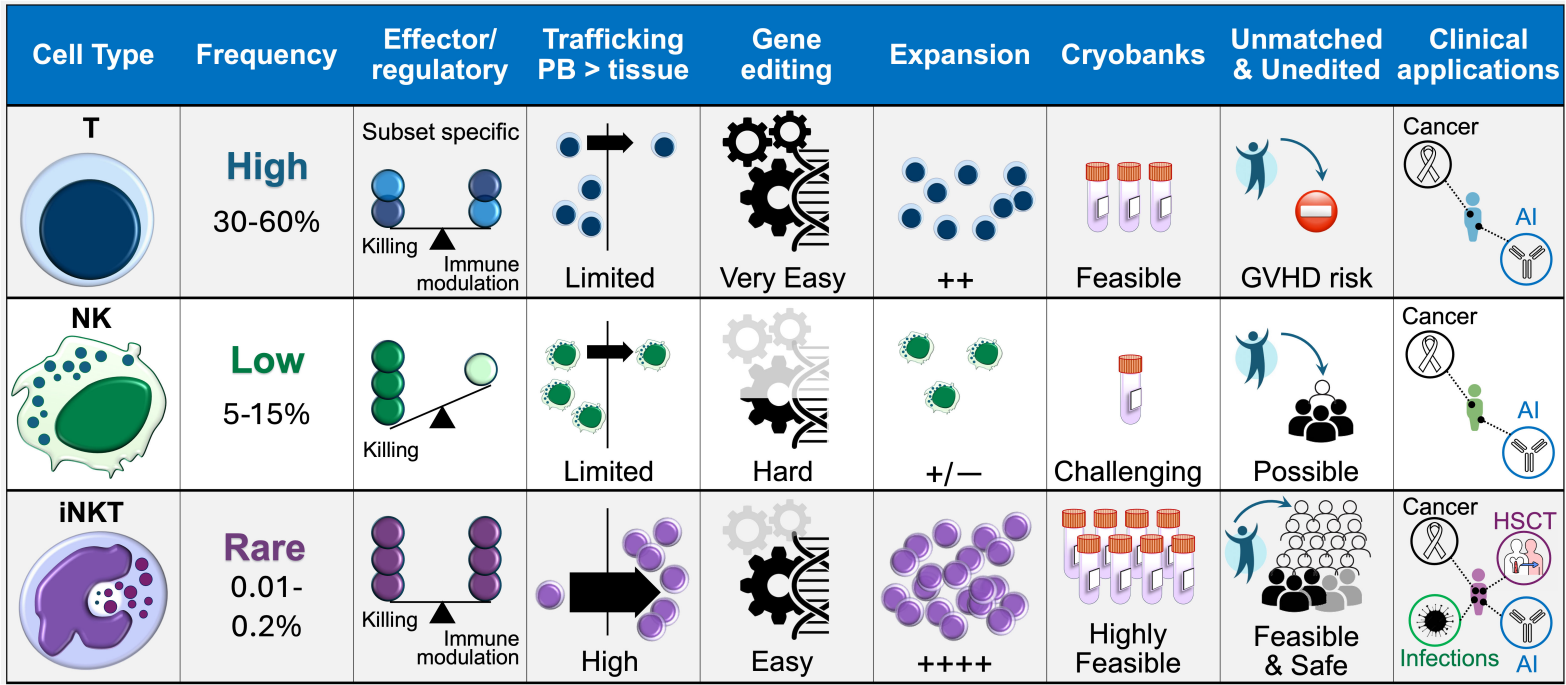
Comparison between T, NK and iNKT cell platforms. The biological properties of iNKT cells, along with their amenability to CAR engineering, *in vitro* expansion, and off-the-shelf use, lend themselves to clinical application in any patient regardless of MHC match and across a range of pathological settings, potentially enhancing the feasibility and accessibility of adoptive cell therapies. AI, autoimmune conditions.

A second major advancement in iNKT therapy is the shift toward allogeneic, rather than autologous, approaches to exploit the full functionality of healthy iNKT cells that were never exposed to the suppression by tumors and the toxicity of cancer treatments (**Table 2**). Unlike conventional T cells, iNKTs and CAR-iNKTs do not cause GVHD and can actively suppress alloreactive responses. Moreover, despite their rare frequency in the circulation, iNKT and their engineered products exhibit a unique potential for substantial and sustained antigen-specific proliferative responses (62, 103), making them ideal for streamlining manufacturing and enhancing feasibility of *off-the-shelf* cellular therapies (**Figure 3**).

**Table 2:** Allogeneic iNKT trials.

Trial ID	Disease/ setting	Allogeneic product formulation	Pre-conditioning	Patient Number	Best Response (number of patients)	Other Responses (number of patients)	References
Monotherapy							
Cancer							
NCT04754100	Myeloma	iNKT	No	8	SD (2)	NA	(104)
jRCT2033200116	HNSCC	iPS-iNKT	No	10	SD (5/8*)	NA	(142, 143)
NCT03774654 (ANCHOR)	NHL, ALL, CLL	IL15.CD19-CAR iNKT	Flu/Cy	9	CR (3)	PR (1)	(52)
NCT05487651 (ANCHOR2)	NHL, ALL, CLL	IL15.CD19-CAR iNKT	Flu/Cy	NA	NA	NA	NA
NCT04814004	NHL, ALL, CLL	IL15.CD19-CAR iNKT	No	NA	NA	NA	NA
Infections and related inflammatory conditions							
NCT04582201	ARDS	iNKT	No	20	CR (14)	NA	(50)
IND 29183	ARDS	iNKT	No	1	CR	NA	(50)
IND 30029	ARDS	iNKT	No	1	CR	NA	(60)
Allo-HSCT							
NCT03802695	Haplo-identical donor	Orca-Q, single GVHD prophylaxis	Myeloablative	33	aGFS (28)	RFS -1y (27)	(116–119)
NCT03802695	HLA-identical donor	Orca-Q, NO GVHD prophylaxis	BFT (11), TBI-based (3)	14	aGFS (11)	RFS-1y (12)	(121, 122)
Combinations							
NCT05108623	Solid tumors	αPD-1 plus iNKT	No	14	PR (1)	SD (8)	(130)
NCT06251793	GE tumors	αCTLA4, αPD-1, αVEGF plus iNKT	Paclitaxel	15	NA	NA	(131)
NCT03294954	Neuroblastoma	αTNFα plus CAR iNKT	Flu/Cy	NA	NA	NA	NA

NA, Not Available; SD, stable disease; PR, partial response; CR, complete response; HNSCC, Head and Neck Squamous Cell Carcinoma; NH, Non-Hodgkin Lymphoma; ALL, Acute Lymphoblastic Leukemia; CLL, Chronic Lymphocytic Leukemia; ARDS, Acute Respiratory Distress Syndrome; HLA, Human Leukocyte Antigen; GE, Gastroesophageal; iPS, induced pluripotent stem; GVHD, Graft-versus-host disease; α, anti; Flu/Cy, Fludarabine/Cyclophosphamide; BFT, Busulfan/Fludarabine/Thiotepa; TBI, total body irradiation; *8 out of 10 patients were evaluable at the time of the report; aGFS, acute-GVHD-free survival; RFS, relapse-free survival.

In trial NCT04754100, donor-derived, pure, unedited iNKT cell products, manufactured and cryopreserved ahead of demand, were administered to unrelated and non-lymphodepleted patients with relapsed/refractory multiple myeloma, known to express CD1d (104). Despite relatively high cell doses (up to total 1×10^9^), no GVHD, CRS, or ICANS was observed. Stable disease was reported in two of eight treated patients, including one with a sustained reduction of over 50% in bone marrow malignant cells despite prior refractoriness to six lines of treatment, including anti-CD38, PD-1, and SLAMF7 monoclonal antibodies (104).

Allogeneic iNKTs are now being explored in engineered settings. In trial NCT00840853, CD19-CAR-iNKT were co-engineered with IL-15 and shRNAs targeting β2-microglobulin and CD74, established strategies to downmodulate MHC class I and II molecules and promote immune evasion, and administered alongside a standard lymphodepleting regimen. Preliminary data showed that as low as 1 ×10^7^ CD19-CAR-iNKTs/m^2^ induced complete remission in patients with refractory B cell malignancies, which included one acute lymphoblastic leukemia and three non-Hodgkin lymphoma (NHL) patients respectively (52). Donor iNKTs remained detectable for five weeks, a preliminary observation that raises as-yet unresolved questions about the role of iNKT cells in immune rejection, and the potential for their immune-tolerizing capabilities to counteract elimination of donor-derived CAR-iNKTs themselves. To date, this aspect of iNKT biology remains largely uncharted. It is also unclear whether long-term functional persistence of iNKTs is required to sustain remission, or if transient restoration of anti-tumor immunity may be sufficient in some patients. Results from this and future clinical trials using engineered allogeneic iNKTs will provide critical insights into these important questions.

To further advance off-the-shelf iNKT therapies, researchers are also exploring induced pluripotent stem cell (iPS)- and hematopoietic stem cell (HS)-derived iNKTs (105). In a first-in-human trial (jRCT2033200116), iPS-iNKTs are being administered into tumor-feeding arteries of patients with advanced recurrent/refractory head and neck squamous cell carcinoma (HNSCC) (106). The primary endpoint is to determine the safety profile and dose-limiting toxicity with infusions of up to 1×10^8^ iPS-NKT cells/m^2^ without prior lymphodepletion (106), laying the foundation for clinical development of enhanced CAR iPS-NKTs (107, 108). Similarly, CAR HS-iNKTs are in preclinical development, with studies indicating that they retain the innate invariant NKT cell’s TCR, NK-like, and CAR effector functions of PBMC-derived iNKTs (109, 110). Both PBMC-derived and stem-cell-induced iNKTs boast exceptional expandability, enabling the production of many billions of cells in a single manufacturing run. However, a side-by-side comparison suggests that HS-iNKTs are skewed towards NK-like differentiation with more prominent direct cytotoxicity, and are phenotypically less immunogenic, which could enhance resistance to host immune rejection (109).

Altogether, these studies represent a stepping-stone towards the development of *allo-resistant*, off-the-shelf cells. While iNKTs’ ability to prevent their own rejection remains hypothetical, clinical and preclinical studies with allogeneic donor cells demonstrate their versatility and scalability for off-the-shelf cancer immunotherapy. The achievement of complete remissions in refractory B malignancies marks a major milestone (52). With iPS-NKT therapies already in the clinic and HS-iNKT therapies on the horizon, the field is rapidly advancing. These next-generation approaches promise more accessible, potentially more effective treatments, positioning the iNKT platform at the forefront of cancer immunotherapy with growing clinical evidence of their benefit.

## 5 Donor iNKTs show life-saving potential in acute inflammatory and infectious diseases

iNKTs play multiple, pivotal roles in the rapid clearance of pathogens, and they are critical in acute viral infections, where low iNKT numbers often correlate with severe outcomes (42, 46). While CD1d and glycolipid agonists have been extensively explored as vaccine adjuvants and potential treatments for chronic viral infections, direct administration of donor iNKTs could offer a more powerful strategy in inducing rapid remission in critically ill patients, overcoming limitations of patient iNKT’s low numbers and dysfunctions. Additionally, off-the-shelf availability without the need for prior chemotherapy or donor-recipient MHC matching, combined with iNKT’s innate-like, fast-acting function, make them particularly well suited for emergency interventions.

In trial NCT04582201, a cryobank of unmodified, allogeneic iNKT cells expanded from healthy donors enabled the treatment of twenty-one critically ill patients with acute respiratory distress syndrome (ARDS), including those on mechanical ventilation (50). A single infusion of donor-derived iNKTs led to a significant survival benefit (70% compared to 10-39% in controls), with some patients able to be extubated within 24 hours from iNKT infusion. The treatment also markedly reduced secondary infections, with no treatment-related toxicity observed even at doses as high as 10^9^ cells. These impressive outcomes likely stemmed from iNKTs’ dual antiviral and immunomodulatory effects, effectively reversing immune exhaustion, mitigating cytokine storms, and restoring immune homeostasis. Further underscoring their clinical potential, a recent case report described a dramatic recovery in an immunosuppressed kidney transplant patient who developed severe COVID-19, progressing to respiratory failure requiring mechanical ventilation and extracorporeal membrane oxygenation (ECMO) (60). Complications included severe disseminated intravascular coagulation (DIC), life-threatening gastrointestinal and airway bleeding necessitating transfusions, and renal failure requiring continuous renal replacement therapy. Remarkably, a single infusion of 1 × 10^9^ iNKT cells, infused under emergency authorization (IND 30029), led to rapid and complete recovery: the patient was weaned off oxygen, dialysis, and returned to baseline renal function with full independence in daily activities.

Collectively, these findings represent a milestone in off-the-shelf allogeneic cell therapy, demonstrating the feasibility, safety, and efficacy of unedited iNKTs in acute infectious settings where conventional MHC-restricted T cell therapies are neither indicated nor feasible. The potential for redosing iNKTs (possibly optimally from unrelated donors) without sustained alloantibody responses (50) further supports their suitability for repeated administration in patients facing recurrent infections, paving the way for broader clinical applications in critical care medicine.

## 6 iNKTs cells pave the way for allo-transplantation without immunosuppressive agents

Both donor and recipient iNKTs can play a critical role in suppressing alloreactive T cells responsible for GVHD. For example, clinical studies and experimental murine models of acute GVHD demonstrated that total lymphoid irradiation (TLI) preconditioning may promote the relative expansion of endogenous radio-resistant, Th2-polarized iNKTs, leading to the suppression of donor alloreactive T cells and activation of regulatory T cells (Tregs) (57–59). Similarly, donor iNKTs were shown to alleviate experimental GVHD in models of human alloreactivity and murine allogeneic transplantation (56, 111). As such, there is increasing interest in iNKT-based therapies for GVHD treatment.

Beyond their suppressive effects, iNKTs also play an active preventive role in GVHD, as demonstrated by findings in allo-HSCT recipients, where a lower risk of acute GVHD correlated with higher numbers of CD4^-^ iNKT cells in donor grafts (56, 57), as well as faster and more robust post-transplant recovery of circulating iNKT cells (112–114). In pediatric haploidentical allo-HSCT, higher iNKT cell counts were associated not only with GVHD protection, but also with a reduced risk of leukemia relapse (115), further supporting the use of iNKTs as an anti-GVHD prophylaxis after allo-HSCT. The ability of iNKT to control pathogen infections (42, 46) provide additional rationale for their use in HSCT, where acute viral, bacterial and fungal infections and reactivation of latent virus infections, are frequent and at high risk of preventing engraftment as well as being fatal.

A Phase 1 study (NCT03802695) is evaluating the safety, tolerability, and efficacy of engineered donor grafts, termed Orca-Q grafts, in which CD34^+^ hematopoietic stem cell products are enriched in donor iNKTs, along with other T cell subsets prior to infusion (116–119). Among thirty-three recipients of haploidentical HSCT who underwent myeloablative conditioning and received only minimal GVHD prophylaxis (standalone tacrolimus without post-transplant cyclophosphamide), no cases of grade 4 acute GVHD were observed (120). Grade 2–3 acute GVHD occurred in only five patients, and no moderate-to-severe chronic GVHD was reported at ~1-year median follow-up compared to a historical control cohort with a 24–33% incidence of moderate-to-severe chronic GVHD. Additionally, one-year relapse-free survival reached 82%, highlighting the therapeutic promise of iNKT-enriched donor grafts (76). In an expansion arm of the same study, Orca-Q was administered without any GVHD prophylaxis (121, 122). This cohort differed from the previous one in that patients and donors were HLA-identical, and myeloablative conditioning was either busulfan/fludarabine/thiotepa (BFT; eleven patients) or total body irradiation-based (TBI; three patients). In the BTF group, only two had acute GVHD (grade 2), none developed chronic GVHD, and only one patient experienced relapse. At one year, overall survival (OS), relapse-free survival (RFS), and GVHD/relapse-free survival (GRFS) were all 90%. In the TBI group, one case of grade 3 acute GVHD and one of mild chronic GVHD were observed, leading to an 85% OS and RFS, and a 77% GRFS. These results highlight the potential of iNKT-enriched donor grafts to act as a bridge to faster immune reconstitution, reducing the need for anti-GVHD immune suppression while preserving GVT effect. Outcomes could be further improved in combination with iNKT-tailored preconditioning regimens.

Building on this momentum, a clinical trial at University Hospital Pilsen, Czech Republic, set to begin in 2026, will evaluate the feasibility and safety of third-party iNKT products as a part of GVHD prophylaxis in allo-HSCT recipients (123). Unlike prior studies in the transplant setting that have relied on donor at least partially matched iNKT cells, this trial will investigate the prophylactic infusion of third-party iNKT cells, further highlighting their unique versatility beyond conventional therapeutic applications. Similarly, MiNK Therapeutics, in collaboration with the University of Wisconsin, is developing an allo-iNKT platform for the prevention (as well as the treatment) of GVHD (124, 144). Preclinical studies in mice support this approach (53), demonstrating that third-party iNKTs can provide GVHD protection. By eliminating the requirement for donor-matched iNKTs, this strategy could significantly expand the feasibility and availability of iNKT therapies for both GVHD prophylaxis and broader applications in allo-HSCT.

## 7 Rational combinations with iNKT cells

Historically, the first trials exploring combinatorial strategies involving iNKT therapy employed DCs pulsed with KRN7000, a synthetic αGalCer derivative and potent iNKT agonist that is effectively presented by DCs and induces production of both Th1 and Th2 cytokines (UMIN000000722 (125), UMIN000000852 (126). Overall, eight out of eighteen patients with recurrent/refractory HNSCC showed significant tumor shrinkage within four to five weeks following infusion of iNKTs via tumor-feeding arteries in combination with one or two submucosal doses of KRN7000-pulsed DCs. Nine patients maintained stable disease, and all but three exhibited systemic immune responses and tumor-infiltrating lymphocyte (TIL) expansion, with no severe side effects. These studies demonstrated the feasibility of iNKT combinations to achieve superior activation, while preserving a safe profile. Variations in the lipid chemistry and formulation as well as the choice of specific APCs may in future enable tailored approaches to distinct clinical settings. For example, unlike aqueous KRN7000, liposomal formulations preferentially target B cells and skew iNKT responses toward allotolerizing cytokines, raising the possibility that they could be leveraged to promote both persistence of allogeneic iNKTs and CAR-iNKTs and optimal activation (62, 127, 128). Pharmacologic immune modulation in addition to KRN7000 has also been proposed, e.g., aimed at upregulating CD1d transcriptionally and epigenetically on CAR-iNKT targets to enhance killing, or co-administering low-dose lenalidomide to augment iNKT immunoadjuvant responses (129).

Checkpoint inhibitors have also been explored in combination with adoptively transferred iNKTs from healthy donors in relapsed or refractory solid tumors (NCT05108623) (130). A patient with refractory gastric cancer who had previously failed anti-PD-1 therapy demonstrated significant tumor regression after receiving a single iNKT cell infusion in combination with anti-PD1, with evidence of local immune activation and CD8^+^ T-mediated adjuvant effects (51). These clinical observations support the potential of iNKT-based therapies to overcome immune exhaustion in the TME. Additionally, they provide the rationale for using unmodified iNKT and checkpoint inhibitor combinations, even in CD1d-negative cancers, as an adjuvant strategy to overcome T and NK cell exhaustion and transform the TME into an immunologically “hot” ecosystem that supports continued tumor recognition and killing by endogenous cytotoxic cells. A single-arm phase 2 trial at Memorial Sloan Kettering (NCT06251793) is evaluating allo-iNKTs in combination with anti-CTLA4 (botensilimab), anti-PD1 (balstilimab), anti-VEGF (ramucirumab), and paclitaxel in patients with advanced refractory gastro-esophageal adenocarcinoma. Early results from fifteen patients suggest promising synergy between allo-iNKTs, checkpoint inhibitors, and sequential chemotherapy (131). Systemic increase in interferon-gamma correlated with immune expansion and T cell memory response in the circulation, and T cell infiltration and cross-presentation at the tumor site, all of which are known biomarkers of clinical efficacy and could predict durable clinical responses in otherwise unresponsive solid tumors. Notably, paclitaxel was administered after iNKTs and checkpoint inhibitors, preserving iNKT-mediated immune priming: a benefit that would have been likely lost with pre-transfer lymphodepletion.

**Figure 4:**
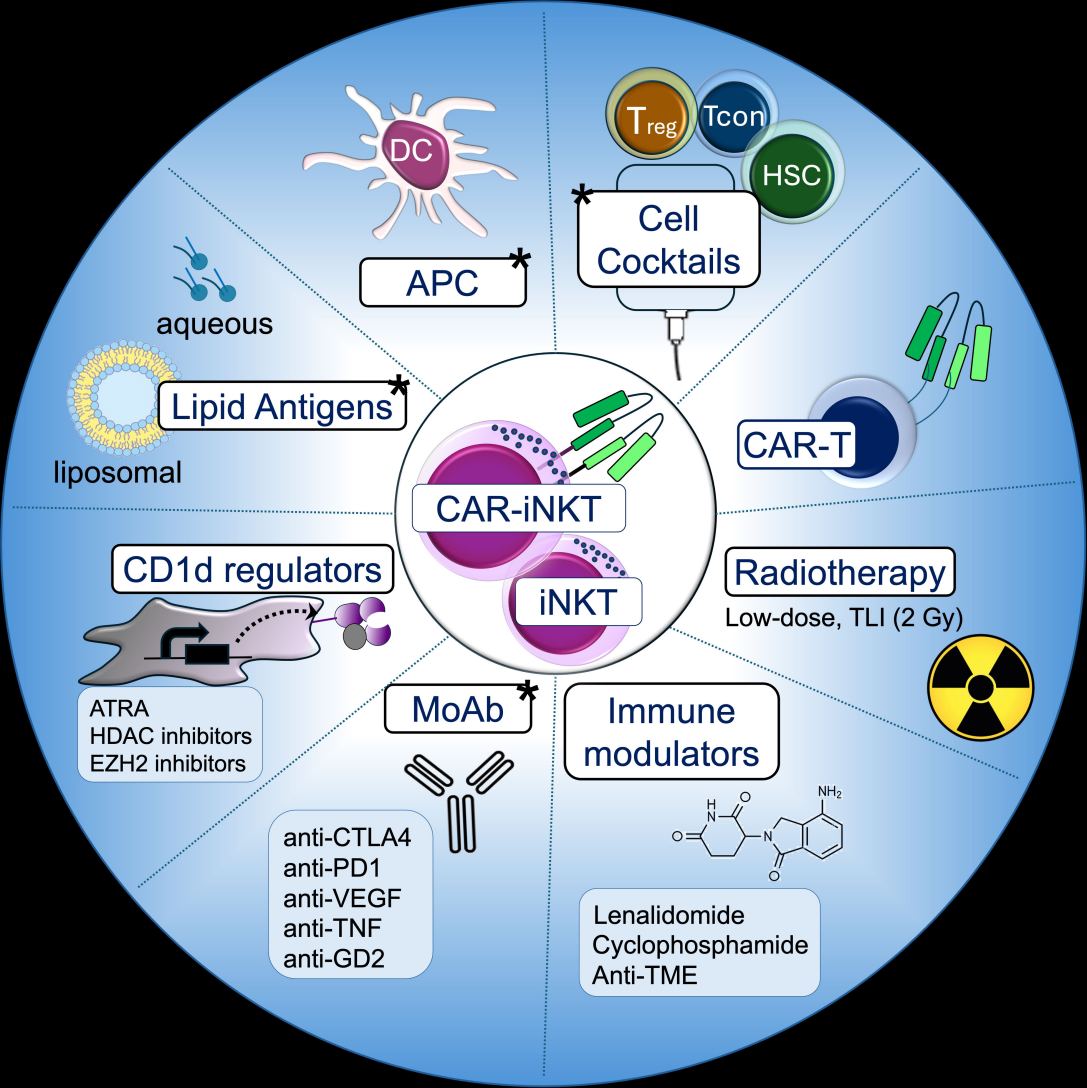
iNKT-based combinations. Strategies involving iNKT and CAR-iNKT cells that have been proposed and clinically tested (marked with a star).

Other monoclonal antibodies have been proposed in combination with CAR-iNKTs. In NCT03294954, GD2-CAR-iNKTs are infused with etanercept, an anti-TNF antibody, based on the observation that TNF promoted tumor progression while inhibition of TNF signaling improved outcomes in murine xenograft models of neuroblastoma (132). Additionally, anti-GD2 monoclonal antibodies are being explored in combination with iPS-derived NKTs to leverage their antibody dependent cellular cytotoxicity (ADCC) against neuroblastoma cells and enhance cytotoxicity through CD16-mediated degranulation and cytokine production (133). Collectively, these studies demonstrate the growing potential of rational combinations not only to enhance iNKT therapies but also overcome barriers to the clinical success of current treatment options in cancer and transplant settings (**Figure 4**).

## 8 Conclusions and prospects

These are transformative times for iNKT cell-based therapies. Since their discovery about 40 years ago, these unique cells have emerged as a powerful tool against a range of currently incurable diseases. Around 200 patients treated with iNKT cell-related therapies have been reported to date, including 129 patients with cancer and severe inflammatory conditions who received autologous or allogeneic iNKT cells, while the remainder were treated with either αGalCer-based approaches or iNKT-enriched cell products. Strong evidence supports that iNKT-based approaches are practical, and that large-scale iNKT cell manufacturing is feasible with promising signs of deep and durable remissions. Some patients with refractory solid tumors achieved complete remissions, while others with acute infection-related life-threatening inflammation were cured. Clinical success was attained with both autologous and allogeneic products, and while further optimizations will be instrumental in realizing their full curative potential, off-the-shelf iNKT therapies appear to be a feasible goal in the next few years.

As clinical trials progress, we see fundamental questions being addressed with important takeaways that will inform future cell therapy studies:

1. How to best harness iNKT cell products and their multiple beneficial activities (anti-pathogen, immunomodulatory, promotion of engraftment and GvHD suppression)? Should persistence be prioritized, or is their ability to restore immune homeostasis sufficient for durable responses? While acute infections and immune-mediated complications may resolve without long-lived donor cells (NCT04582201, IND 30029), the potential of iNKT cells to reverse immune exhaustion and ignite endogenous anti-tumor responses raises the possibility of lasting remissions, even without continuous patrolling by donor-derived iNKTs (NCT05108623). Ongoing and future trials in cancer patients will provide valuable insights into the role of long-lasting immunoadjuvant effects over iNKT persistence and direct immunosurveillance in durable remissions and possibly cures.

2. Given the feasibility of off-the-shelf iNKT products, should customized products, i.e., tailored to different disease settings and patient needs, be employed? Ongoing molecular studies are shedding light on human iNKT heterogeneity within and across individuals, refining our understanding of distinct subsets and their therapeutic roles (NCT03294954 and (62, 98, 134–136). Donor-specific iNKT biomarkers of survival and immune fitness were found to correlate with better clinical outcomes (NCT03294954 and (98, 134, 135), suggesting an intriguing potential for predicting optimal cellular products to ensure maximal therapeutic effect. With the increasing use of single-cell sequencing technologies, novel biomarkers of function could soon guide both donor selection and the customization of cellular products. Additionally, trials in canine cancer patients have emerged as a valuable preclinical platform to refine iNKT cell therapies in a parallel patient population with tumors and immune system that closely mirror those of humans (62). These studies serve as a critical bridge between mouse and human, enabling the selective advancement of the most promising iNKT therapies to the clinic, with designs best suited to patient needs and disease contexts.

3. Regarding the need of bespoke approaches, should iNKT-guided schedules be designed and administered using *ad hoc* preconditioning regimens based on iNKTs’ unique mechanisms? Thus far, PBMC-derived allogeneic CAR-iNKTs have followed conventional CAR-T cell paradigms, including MHC editing and lymphodepletion typically required to counteract T cell rejection (NCT00840853, and (52, 137)). However, some allogeneic iNKT and iPS-iNKT products have been administered without prior MHC editing or intensive preconditioning, capitalizing on iNKT-specific mode of action (jRCT2033200116, NCT04754100, NCT05108623, NCT06251793). Trials and correlative studies in COVID-19 patients with severe ARDS (50) and parallel studies in canines (62) support a path toward such administration strategy, informed by iNKTs’ intrinsic properties rather than conventional T-cell approaches.

4. Last, iNKT safety profile makes them ideal for combinatorial strategies, integrating with pharmacologic agents, biologics, and even other cell therapies. Learning from the success of polytherapy regimens that improved clinical outcomes of cancer patients compared to standalone monotherapies (138–140), will the optimal iNKT therapy be a multimodal platform incorporating synergistic compounds, biological agents and other cell types? The emerging evidence that iNKTs can mediate durable donor cell engraftment with simultaneous anti-GVHD and pro-GVT effects (121) may offer unprecedented opportunities to combine iNKT cells with multiple allogeneic cell types: bulk T, selected αβ or *y*ð T, NK, macrophages and other APCs, and even non-hematopoietic stem cell and progenitors, whether unmodified or engineered with CARs, TCRs and other molecules, and from the same or different donors. The projected clinical impact is far-reaching, with the potential to expand the versatility, feasibility, and efficacy of cellular therapies by harnessing the complementary therapeutic profiles of distinct cell populations across a range of diseases while minimizing the risk of severe toxicities.

While future trials are anticipated to address these points, iNKT present two significant advantages over conventional T cell therapies, supporting their broad clinical implementation. Firstly, both allogeneic and stem cell-derived iNKTs offer exceptional scalability, with evidence of over a million-fold expansion in less than one month of *ex-vivo* culture, far surpassing the capabilities of conventional T cell-based products (62, 88). One production round can yield thousands of billions of cells (> 1000 doses), enabling the creation of cost-efficient, universal cell banks. This could revolutionize accessibility, providing ready-to-use, off-the-shelf therapies that transcend geographical and logistical barriers. Secondly, iNKT cells represent a uniquely versatile platform, with applications spanning cancer, infections, and immune-mediated disorders associated with promising clinical evidence of efficacy. Unlike conventional adoptive T and NK cell therapies, iNKT cells act rapidly, exert both effector and immunomodulatory functions, and may not require chemotherapy preconditioning, vastly expanding their therapeutic reach.

With these strengths, iNKT cell therapies are poised to gain significant traction in the coming years, offering an excellent opportunity to reshape treatment landscapes and deliver breakthrough therapies for patients who currently have no options. As the field advances, the next decade may mark the tipping point where iNKT cells transition from a promising innovation to a cornerstone of cellular immunotherapy.
